# MicroRNAs in Vascular Biology

**DOI:** 10.1155/2012/794898

**Published:** 2012-09-26

**Authors:** Munekazu Yamakuchi

**Affiliations:** Department of Medicine, Aab Cardiovascular Research Institute, University of Rochester School of Medicine and Dentistry, 601 Elmwood Avenue, Box CVRI, Rochester, NY 14642, USA

## Abstract

Vascular inflammation is an important component of the pathophysiology of cardiovascular diseases, such as hypertension, atherosclerosis, and aneurysms. All vascular cells, including endothelial cells (ECs) and vascular smooth muscle cells (VSMCs), and infiltrating cells, such as macrophages, orchestrate a series of pathological events. Despite dramatic improvements in the treatment of atherosclerosis, the molecular basis of vascular inflammation is not well understood. In the last decade, microRNAs (miRNAs) have been revealed as novel regulators of vascular inflammation. Each miRNAs suppresses a set of genes, forming complex regulatory network. This paper provides an overview of current advances that have been made in revealing the roles of miRNAs during vascular inflammation. Recent studies show that miRNAs not only exist inside cells but also circulate in blood. These circulating miRNAs are useful biomarkers for diagnosis of cardiovascular diseases. Furthermore, recent studies demonstrate that circulating miRNAs are delivered into certain recipient cells and act as messengers. These studies suggest that miRNAs provide new therapeutic opportunities.

## 1. Introduction

Atherosclerosis is the major cause of death in western countries; atherosclerosis leads to cardiovascular diseases such as peripheral artery disease, acute coronary syndromes, and aneurysms [1]. The pathology of atherosclerosis develops in discrete stages: normal vessel wall, fatty streaks, atherosclerotic plaques, and ruptured plaques with thrombosis. The cellular and molecular events that lead to these pathological changes are well studied and include endothelial dysfunction, monocyte adherence and entry into the vessel wall, monocyte development into foam cells, smooth muscle cell migration and proliferation, and platelet adhesion and aggregation [2, 3]. Vascular inflammation drives the entire process of atherogenesis [4, 5]. Healthy endothelial cells (ECs) control vascular tone, limit vascular smooth muscle cells (VSMCs) proliferation, inhibit leukocyte adherence, and block thrombosis [6]. ECs release a set of factors that promote vascular homeostasis, including nitric oxide and prostacyclin [7]. However, a variety of vascular injuries destroy the ability of the endothelium to protect the vessel wall. Diabetes, hypertension, hyperlipidemia, and smoking can damage ECs [8–10]. Dysfunctional ECs make less nitric oxide and less prostacyclin [11, 12]. Furthermore, injured ECs express proinflammatory soluble and membrane bound mediators, including chemokines and p-selectin and vascular cell adhesion molecule-1 (VCAM-1), which increase leukocyte trafficking, as well as von Willebrand factor (VWF) which promotes thrombosis [13]. Several inflammatory pathways in the vasculature have been well defined [14]. For example, oxidized LDL can activate the nuclear factor *κ*B (NF-*κ*B) pathway, inducing the expression of a set of inflammatory genes [15]. Also, angiotensin II (AngII) activates Ets-1, a key endothelial transcription factor, leading to expression of VCAM-1 by several stimuli [16].

Recent work by several investigators has revealed that microRNAs (miRNAs) can also control vascular inflammation. This paper summarizes the role of miRNAs in vascular inflammation and highlights recent evidence that circulating miRNAs are not only biomarkers for disease but also serve as cell-to-cell messengers.

## 2. Biogenesis of miRNAs (See Figure 1)

MicroRNAs (miRNAs) are small noncoding RNAs of 18–22 nucleotides in length, which regulate gene expression posttranscriptionally [17–19]. miRNAs regulate diverse biological functions, including cell proliferation, apoptosis, senescence, differentiation, metabolism, tumorigenesis, and developments. Mature miRNAs are generated from primary miRNAs (pri-miRNAs) by two RNase III enzymes—Drosha and Dicer [20]. The Drosha complex processes pri-miRNAs into hairpin miRNA precursors (pre-miRNAs) in the nucleus; then Dicer cleaves these pre-miRNAs into miRNA duplexes in the cytoplasm. Recently it has shown that some miRNA precursors are generated by a Drosha-independent pathway [21]. One strand of this miRNA duplex is incorporated into the RNA-induced silencing complex (RISC) and acts to guide the RISC complex to its targets.

In mammals, mature miRNAs can bind to the 3′ untranslated region (3′-UTR) of target genes by partial complementarity. The interaction of the 5′ end of miRNAs (the seed sequence) with the target mRNA is sufficient to stop translation of target genes. miRNAs limit gene expression by (1) degradation of mRNA or (2) inhibition of translation initiation [19]. More than 1000 miRNAs are encoded in the human genome (http://www.mirbase.org/). Computer algorithms predict that most miRNAs have multiple potential target genes, based on potential interactions between the 3′UTR of mRNA and the miRNA seed sequences. In fact, it is predicted that miRNAs can manage the regulation of at least 60% of protein-coding genes in humans [18, 22].

## 3. Endothelial miRNAs (See Figure 2)

The physiological and pathological roles of miRNAs have been widely studied. Dysregulation of miRNAs cause a variety of diseases, including cancer [23, 24], neuropsychiatric disease [25, 26], diabetes [27], and renal failure [28]. miRNAs expressed in the vasculature play important roles in cardiovascular diseases [29]. A series of miRNAs control inflammation and oxidative stress in vascular cells including ECs, VSMCs, and inflammatory cells [20, 30, 31]. ECs control vascular homeostasis [32, 33]. miRNAs play an integral role in endothelial regulation of vessel function. Elimination of most endothelial miRNAs by knockdown of Dicer in ECs inhibits proliferation and tube formation in vitro [34]. Moreover, EC-specific Dicer knockout mice have impaired blood vessel development [35, 36]. These findings suggest that miRNAs in ECs are indispensable for the maintenance of vascular homeostasis. Which miRNAs are important in ECs and why?

### 3.1. miR-126: A Guardian miRNA in ECs

miRNA profiling data suggest that miR-126 is expressed mainly in ECs and platelets [34, 37, 38]. Interestingly, miR-126 is located in the intron of epithelial growth factor like domain containing protein 7 (EGFL7), an endothelial-specific protein involved in development of the vasculature [39]. During splicing of EGFL7 pre-mRNA, miR-126 is excised. miR-126 itself plays a central role in vascular development. Knockout of miR-126 in mice and zebrafish decreases vascular integrity and impairs proliferation, migration, and angiogenic activity of ECs [40, 41]. miR-126 knockout in mice is partially embryonic lethal, and surviving miR-126 knockout mice have defective cardiac neovascularization after myocardial infarction [41]. miR-126 enhances VEGF signaling by inhibiting Sprouty-related protein (SPRED1) and phosphoinositol-3 kinase regulatory subunit 2 (PIK3R2/p85-beta) to maintain vascular integrity [40–42]. Thus miR-126 acts as a proangiogenic miRNA by increasing PI3K and MAP kinase signaling. During vascular inflammation, miR-126 is involved in suppressing inflammation signals in ECs. Inflammatory cytokines increase a series of adhesion molecules on the surface of ECs. Harris et al. showed that VCAM-1 is a direct target of miR-126. Knockdown of miR-126 promotes leukocyte adherence to ECs by enhancing TNF-*α* stimulated VCAM-1 expression [37].

### 3.2. Senescence Associated miRNAs

Aging is an independent risk factor for cardiovascular disease [47]. Senescent ECs have increased apoptosis, induce inflammation, and have decreased nitric oxide production by endothelial nitric oxide synthase (eNOS), causing endothelial dysfunction, followed by progression of atherosclerosis [48, 49]. In cultured ECs, both replicative senescence and stress-induced premature senescence release proinflammatory mediators and decrease expression of anti-inflammatory proteins such as eNOS [50, 51]. Several miRNAs are identified as senescent associated miRNAs in many cancers and fibroblasts [52–54]. The profiling of miRNAs in senescent human primary ECs shows that a set of miRNAs, such as miR-17-5p, miR-21, miR-216, miR-217, miR-31b, and miR-181a/b, are highly expressed by aging cells [55]. In addition, some miRNAs such as miR-146a are decreased in senescent ECs. miR-146a regulates NOX4, which is one of NADPH oxidase isoforms and contributes to generation of reactive oxidative stress (ROS) [43]. Since ROS promotes ECs senescence [56], miR-146a suppresses senescence by inhibiting NOX4, suggesting that the decrease level of miR-146a in senescent ECs may promote more aging by enhancing NOX4 expression. 

#### 3.2.1. miR-217

miR-217 is minimally expressed in normal ECs, but miR-217 expression increases in senescent cells. miR-217 represses silent information regulator 1 (SIRT1) expression [55]. SIRT1 is a NAD+-dependent deacetylase that control gene expression by deacetylating target proteins. SIRT1 promotes longevity and prevents stress-induced senescence in ECs [57, 58]. SIRT1 controls a variety of transcription factors such as p53, FoxO (forkhead box O), and PGC-1a (peroxisome proliferators activated receptor gamma coactivator-1a). Overexpression of miR-217 decreases SIRT1 expression, which increases acetylation of FoxO1 in young ECs [55]. Since ectopic expression of FoxO1 inhibits ECs migration and tube formation [59], miR-217 blocks angiogenic property in ECs by inhibiting SIRT1-FoxO1 function. Menghini et al. also demonstrated that miR-217 is negatively correlated with SIRT1 expression in human atherosclerotic plaques [55]. These results suggest that miR-217 has an important role in the pathogenesis of atherosclerosis in vitro and in vivo.

#### 3.2.2. miR-34a

miR-34a expression increases in senescent ECs. Ito et al. demonstrated that the expression of miR-34a in heart and spleen are higher in aged mice than in young mice [60]. Ectopic expression of miR-34a induced senescence and cell cycle arrest in ECs. Since SIRT1 has been shown to be a direct target of miR-34a, miR-34a promotes aging of ECs through SIRT1 inhibition. miR-34a also inhibits endothelial progenitor cells (EPC) mediated angiogenesis by induction of senescence [61]. EPCs are involved in new blood vessel formation to maintain ECs homeostasis and the number of EPCs is reduced in atherosclerotic patients [62], indicating that miR-34a may be implicated in the progression of atherosclerosis; however, the relationship between miR-34a and atherogenesis is not defined yet.

#### 3.2.3. miR-21

Several miRNAs including miR-21 and miR-214 are downregulated in senescent human aortic endothelial cells (HAEC) compared with young HAEC [63]. miR-21 regulates cell proliferation by suppressing phosphatase and tensin homolog deleted on chromosome 10 (PTEN), a potent negative regulator of PI3K/Akt signaling pathway. PTEN suppresses Akt signaling, which decreases eNOS activity and PTEN also inhibits VCAM-1 expression in TNF-*α*-stimulated ECs [64], suggesting that miR-21 promotes inflammation in ECs. Several pathological conditions lead to increased miR-21 levels. Shear stress induces miR-21 expression and high miR-21 level is observed in vessels during pulmonary hypertension [65, 66]. miR-21 also contributes to endothelial-to-mesenchymal transition (EndMT). EndMT is a phenotypic change of ECs into fibroblastic cells. Blockage of miR-21 suppresses TGF-*β*-induced EndMT by inhibiting phosphatase and tensin homolog (PTEN) in ECs [67]. Pressure overload of left ventricular in mice increases miR-21 expression and fibroblast markers in cardiac ECs; miR-21 antagomir blocks this effect [67]. These data indicate that miR-21 modulates vascular homeostasis through PTEN and Akt.

### 3.3. Angiogenesis Associated miRNAs

Angiogenesis plays an important role in the development of atherosclerosis [68, 69]. Recent studies identified several miRNAs involved in angiogenesis. These miRNAs are separated in two groups: proangiogenic miRNAs and antiangiogenic miRNAs.

#### 3.3.1. miR-17-92 Cluster

The miR-17-92 cluster is a polycistronic miRNA gene (c13orf25 transcript), containing six tandem stem-loop hairpin structure that produce six mature miRNAs: miR-17, miR-18a, miR-19a, miR-19b-1, miR-20a, and miR-92 [70, 71]. Moreover, two miR-17-92 cluster paralogs exist, miR-106a-363 and miR-106b-25. This miRNA polycistron is functionally categorized into four families: (1) miR-17 family, (2) miR-18 family, (3) miR-19 family, and (4) miR-92 family. The c13orf25 transcript containing miR-17-92 precursor is often elevated in many cancers [72–75]. In ECs, the expression level of miR-17-92 cluster is high [76]. Impaired angiogenic activity by knockdown of Dicer in ECs is rescued by adding individual miRNAs in the miR-17-92 cluster [36]. Bonauer et al. showed that miR-92a in ECs suppresses angiogenesis in vitro and in vivo. Overexpression of miR-92a targets integrin *α*5 (ITGa5) and inhibits angiogenic activity in ECs. Administration of antagomir-92a blocks neovascularization in mouse hindlimb ischemia model and limits tissue injury in myocardial infarction [77]. How do the other miRNAs of these clusters function? Overexpression of miR-17, miR-18a, miR-19a, and miR-20a inhibits endothelial sprouting in vitro. In vivo, inhibition of miR-17 and miR-20a increase the number of lectin-perfused vessels in Matrigel plugs, but knockdown of miR-18a and miR-19a does not [78]. These findings indicate that individual miRNAs in this cluster function as negative regulators of angiogenesis.

#### 3.3.2. miR-23-27-24 Cluster

The miR-23-27-24 clusters are enriched in ECs [79]. There are two highly conserved clusters: an intergenic miR-23a-27a-24-2 cluster and an intronic miR-24b-27b-24-1 cluster [80]. miR-23a is upregulated during hypertrophy by pressure overload or isoproterenol treatment [81, 82]. miR-27 is involved in the initiation and progression of atherosclerosis [83]. miR-27b targets thrombospondin-1 (TSP-1), an endogenous angiogenesis inhibitor [34, 79]. Inhibition of miR-27b reduced in vitro sprout formation [34]. TSP-1 deficiency accelerates atherosclerotic plaque maturation in ApoE knockout mice and dysregulates VSMCs activation in the arterial wall [84, 85]. These results suggest the possibility that miR-27b may promote angiogenesis by TSP-1 inhibition. Another study identified semaphorin 6A (SEMA6A) as a target of miR-27a/b. miR-27a/b negatively regulates ECs sprout formation and knockdown of miR-27a/b blocks embryonic vessel formation in zebrafish [86]. Zhou et al. demonstrated that knockdown of miR-23 and miR-27 impairs sprouting of aorta ring cells, migration, and tube formation of ECs in vitro. miR-23 and miR-27 inhibit expression of sprouty2, semaphorin 6A, and semaphorin 6D, which inhibit angiogenesis. Inhibition of these miRNAs regulates retinal vascular development and choroidal neovascularization in mice [79]. The miR-23-27-24 clusters are therefore involved in angiogenesis and atherosclerosis.

### 3.4. Hypoxia and miRNAs

A constant oxygen supply is necessary to maintain cellular function. Hypoxia triggers special programs to protect cells from irreversible damage [87]. Under normoxia, cells express prolyl hydroxylase domain protein 2 (PHD2 or EGLN), which hydroxylates prolyl residues on hypoxia-inducible factor-1 alpha (HIF-1*α*) [88]. Prolyl hydroxylated HIF-1*α* is immediately degraded by binding to von Hippel-Lindau (VHL) [88]. However, hypoxia suppresses PHD2 activity, stabilizing HIF-1*α*, which then forms heterodimers with its partner hypoxia-inducible factor-1 beta (HIF-1*β*). This complex is translocated into the nucleus and promotes expression of hundreds of hypoxia regulated gene, such as vascular endothelial growth factor (VEGF) [87]. These hypoxia-regulated proteins increase ECs proliferation and migration to promote angiogenesis.

#### 3.4.1. miR-210

Hypoxia induces miR-210 expression in ECs as well as in cancer cells [89]. In cancer, the expression level of miR-210 is correlated with poor survival in cancer patients [90, 91]. Interesting target genes of miR-210 include glycerol-3-phosphate dehydrogenase (GPD1L) [92] and mitochondrial components (NADH dehydrogenase (ubiquinone) 1 alpha subcomplex 4 (NDUFA4) and succinate dehydrogenase complex, subunit D (SDHD)) [90]. Moreover, miR-210 expression is associated with expression of VEGF signaling molecules in clinical breast cancer [93]. In ECs, miR-210 controls the receptor tyrosine kinase ephrin-A3 (EFNA3) that is involved in vascular remodeling. Overexpression of miR-210 increases ECs migration; inhibition of miR-210 decreases ECs tube formation under hypoxia [94]. These data suggest that miR-210 promotes angiogenesis. HIF-1*α* directly binds to hypoxia responsive element (HRE) on the promoter of miR-210, following production of miR-210 transcripts [89].

#### 3.4.2. miR-424 and miR-503

miR-424 and miR-503 are derived from a polycistronic precursor miR-424-503. These miRNAs are induced during monocyte differentiation [95] and myogenesis [96]. miR-503 expression in ECs is upregulated by high glucose or the absence of growth factors [97]. miR-503 targets cdc25a and cyclinE1 (CCNE1) protein [97]. cdc25a is a protein phosphatase that drives cell cycle by activating cyclin-dependent protein kinases (CDKs) and CCNE1 functions as a regulator of CDKs [98]. Both promote ECs proliferation by controlling cell cycle progression [99]. Therefore miR-503 overexpression inhibits ECs proliferation by suppressing cdc25A and CCNE1. miR-503 expression is increased in ischemic adductor muscles of hindlimb ischemia model in streptozotocin-induced diabetic mice and administration of miR-503 decoy to inhibit miR-503 recovers postischemic angiogenesis [97]. miR-424 is also induced by hypoxia in several cell type including ECs [100]. An ubiquitin ligase scaffold protein cullin-2 (CLU2) destabilizes HIF-1*α* to assemble an E3 ubiquitin ligase complex [101]. Hypoxia-induced miR-424 decreases CLU2 protein expression, which in turn stabilizes HIF-1*α* and promotes hypoxia regulated gene expression, which increases proliferation and migration of ECs and angiogenesis in mice [100]. Ghosh et al. also studied the transcriptional mechanism of miR-424. C/EBP*α* levels increase in hypoxic ECs. C/EBP*α* bound with RUNX-1 activates the PU.1 promoter and increased PU.1 then induces the expression of miR-424 [100]. Another group demonstrated unique functions of miR-424 in ECs [102]. VEGF and fibroblast growth factor 2 (FGF2) increase miR-424 and miR-16, and these miRNAs target VEGF receptor 2 (VEGFR2) and FGF receptor 1 (FGFR1) [102]. miR-16 and miR-424 are located in different gene locations but have the same seed sequence, so it is not surprising that miR-16 and miR-424 share the same target genes. In this case, miR-424 overexpression reduces proliferation and migration in ECs [102]. Interestingly VEGF and FGF2 increase mature miR-424, but not pri-miR-424 in ECs, suggesting that increase of miR-424 expression by VEGF and bFGF stimulation are not because the induction of transcription, but due to a positive regulation of miRNA processing from the preexisting primary transcript [102].

### 3.5. Inflammation and miRNAs

Vascular inflammation is an early step in atherogenesis, and many miRNAs are induced in inflamed ECs.

#### 3.5.1. miRNAs Regulating NF-**κ**B-Dependent Pathway

Proinflammatory cytokines (TNF-*α*, IL-1*β*) and LPS increase a set of adhesion molecules in ECs, which recruit inflammatory cells to the site of inflammation [103]. This induction of adhesion molecules is mainly mediated by the NF-*κ*B pathway [104]. Among many different homodimers and heterodimers in the NF-*κ*B/Rel family, the p50/p65 heterodimer is predominant in ECs [105]. In resting ECs, NF-*κ*B binds to I*κ*B protein, an inhibitor protein of NF-*κ*B, and localized in the cytoplasm [106]. Once ECs are activated, I*κ*B kinase (IKK) complex is phosphorylated, which rapidly degrades I*κ*B*α* by the 26S proteasome. This leads to translocate NF-*κ*B heterodimers into nucleus immediately, following the induction of the number of inflammatory response genes [107].

Sun et al. showed that TNF-*α* treatment decreases miR-181b expression in ECs [108]. Overexpression of miR-181b blocks the induction of adhesion molecules, such as VCAM-1, in vitro and in vivo. Systematic administration of miR-181b mimics reduces leukocyte accumulation and ECs activation in LPS-induced lung injury. Sun et al. demonstrated that miR-181b targets importin-*α*3, which is required for nuclear translocation of NF-*κ*B, suggesting that the inhibitory effects of miR-181b on TNF-*α* induced expression of adhesion molecules are mediated by repression of NF-*κ*B nuclear translocation. 

Fang et al. demonstrated that miR-10a/b expression is lower at athero-susceptible arterial sites compared with athero-protected sites in dorsal thoracic aorta from swine [109]. Fang et al. then showed that miR-10a directly inhibits two key molecules of I*κ*B*α* degradation, mitogen-activated kinase kinase kinase 7 (MAP3K7 or TAK1) and beta-transducin repeat-containing gene (betaTRC). Knockdown of miR-10a decreases the expression of MAP3K7 and betaTRC, which upregulates phosphorylation of I*κ*B*α*, causing more nuclear transport of NF-*κ*B p65 and upregulation of the inflammatory cytokines such as MCP-1, IL-6, IL-8, VCAM1, and E-selectin (SELE) [109]. This suggests that miR-10a contributes to the regulation of inflammatory response through NF-*κ*B pathway in ECs.

#### 3.5.2. miR-31 and miR-17

miR-31 and miR-17 are induced by TNF-*α* in human umbilical cord endothelial cells (HUVEC) and miR-31 regulates SELE and mIR-17-3p targets intercellular adhesion molecule 1 (ICAM1) in ECs [110]. Both miRNAs control neutrophil adhesion to ECs in vitro, suggesting that miR-31 and miR-17-3p limit vascular inflammation by regulating the expression of adhesion molecule [110].

#### 3.5.3. miR-155

TNF-*α* treatment of ECs induces other miRNAs such as miR-155, miR-221, and miR-222 [111]. These miRNAs are enriched in ECs and target Ets-1, a key endothelial transcription factor [111]. Stimulation with angiotensin II increases downstream genes of Ets-1, including VCAM1, monocyte chemotactic protein 1 (MCP1), and fms-related tyrosine kinase 1 (FLT1); overexpression of miR-155 partially restores this effect, suggesting that miR-155 regulates adhesion of T cells to activated ECs. Angiotensin II type 1 receptor (AT1R) is another target of miR-155 [112]. Interestingly, the human AT1R contains a +1166 A/C polymorphism, which enhances AT1R activity [113]. Since this +1166 A/C mutation destroys miR-155 binding element (the seed sequence), this mutation often maintains high AT1R activity [112].

#### 3.5.4. miR-221 miR-222

TNF-*α* increases miR-221/222 expression in ECs. Dicer knockdown enhances eNOS expression in HUVEC, and miR-221 and miR-222 overexpression rescues the enhanced eNOS suppression [20, 35]. Of note, the 3′UTR of eNOS has no target sequence for miR-221/222, suggesting that this regulation is indirect. miR-221/222 inhibits proliferation and migration of ECs [20, 35]. In contrast, miR-221/222 increases VSMCs proliferation and migration [114]. Liu et al. have shown that miR-221/222 targets p27 (Kip1) to suppress endothelial proliferation and growth of VSMCs is promoted by inhibiting c-kit [114]. These opposing cellular effects of miR-221/222 are observed in vivo. miR-221/222 increases neointimal growth but decrease reendothelialization in balloon-injury rat carotid artery model [114].

### 3.6. Kruppel-Like Factors and miRNAs

Krüppel-like family of transcription factor, the zinc finger family of DNA-binding transcription factor, is regulated by several stimuli such as laminar flow and statins in ECs [115]. Kruppel-like factor 2 (KLF2) and KLF4 are implicated in protection of atherogenesis through anti-inflammatory and anticoagulant pathways [116, 117]. Especially KLF2 plays a pivotal role in endothelial biology [117]. KLF2 inhibits cytokine-mediated induction of VCAM-1 and SELE expression, resulting in decreasing inflammation in ECs [118]. KLF2 induces thrombomodulin (TM), a cell surface factor involved in antithrombotic function on the surface of ECs [119]. KLF2 also induces eNOS expression and activity to maintain vasoreactivity and vascular tone [115].

miR-92a negatively regulates KLF4 and KLF2 expression in arterial endothelium [120, 121]. miR-92a, a member of the miR-17-92 cluster, has been identified as an endogenous repressor of angiogenesis (see Section 3.3.1). Overexpression of miR-92a inhibits the expression of eNOS and TM, downstream molecules of KLF2, and administration of miR-92a into mice decreases the expression of KLF2 and eNOS in the arteries [120]. Fang and Davies also demonstrated that atheroprone flow increases the interaction between miR-92a and KLF2 mRNA with Ago proteins, one of the major RNA induced silencing complex, indicating direct evidence that miR-92a regulates KLF2 expression. miR-92a regulates KLF4 expression as well as KLF2 [121]. TNF-*α* increases the expression of Monocyte chemotactic protein 1 (MCP-1), VCAM-1, and SELE in human aortic endothelial cells (HAEC). Fang and Davies demonstrated that knockdown of miR-92a partially suppresses these TNF-*α*-induced endothelial inflammatory mediators through KLF4 and miR-92 knockdown inhibits TNF-*α*-induced leukocyte adhesion to ECs in vitro [121]. These findings suggest that miR-92a in ECs acts as an atheroprotective miRNA by regulating KLF2 and KLF4.

## 4. The Communication of miRNAs between Cells

Human studies have revealed a set of miRNA in blood, joint fluid, and other extracellular locations [122, 123]. Extracellular miRNAs have been used as biomarkers to classify diseases and progression of diseases [124, 125]. Recent studies have revealed that miRNAs also serve as messengers between cells [126–128].

### 4.1. miRNA Secretion (Figure 3)

How do miRNAs exit cells? One mode is by passive leakage from necrotic or apoptotic cells [129]. The other mode is by active secretion from living cells within microvesicles (MVs) or in RNA-lipid/protein complexes [130]. Cytokines or shear stress induce ECs-derived MVs release [131]. In response to these stresses, ECs release three types of MVs: exosomes, microparticles, and apoptotic bodies [132]. Exosomes, lipid bound particles about 30–100 nm in size, are generated through the endosomal pathway from multivesicular bodies (MVB), and then secreted by the fusion of endosome and plasma membrane [133]. Microparticles are released by budding from the outer layers of plasma membranes, and their size is larger than exosomes (100 nm–1 *μ*m) [134]. The much larger size of apoptotic bodies, about 1–3 *μ*m in size, contains miRNAs, DNA, and histones [135]. Apoptotic bodies are released by ECs in atherosclerotic lesions and can fuse to other vascular cells, delivering their contents [128]. Some miRNAs are incorporated into RNA-binding proteins such as Argonaute 2 or nucleophosmin 1 (NPM1) and high-density lipoprotein (HDL) and exist as MVs-free conditions [32, 33, 136]. However, the function of these extracellular miRNAs complexes is still unclear. How are miRNAs packaged into MVs? Kosaka et al. raised one possible answer. Neutral sphingomyelinase 2 (nSMase2) controls ceramide biosynthesis and inhibition of nSMase2 by GW4869 or a silencing RNA decreases secretion of miRNA [137], suggesting that ceramide pathway is involved in MVs secretion.

MVs protect miRNAs from degradation [20, 138]. Naked extracellular miRNAs are immediately degraded by ribonuclease (RNase) [122]. MVs are released into microenvironments near their origin and can be detected in plasma, urine, bile, ascites, cerebrospinal fluid, and breast milk [132]. Circulating miRNAs are also detected in body fluids, such as serum, plasma, urine, and saliva [124, 139–141]. Previous studies demonstrated that MVs play important roles in diverse vascular events. MVs derived from ECs and platelet are elevated in hypertensive patients, suggesting that pressure induced activation of ECs and platelets increase MVs productions [142]. Human atherosclerotic plaques contain a lot of microparticles, which comes from other origins such as platelets, ECs, and monocytes [143, 144]. Platelet MVs and macrophage MVs accumulate in the lipid core of atherosclerotic plaques [145]. Moreover, MVs affect the progression and development of human atherosclerotic lesions by transferring adhesion molecules and cytokines [146]. Interestingly, chronic treatment with antioxidants decreases ECs-derived MVs in patients with heart failure [147]. These reports suggest that molecules including miRNAs inside MVs can regulate functions of recipient cells.

### 4.2. Biomarkers

A variety cells secrete miRNAs, including T cells, monocytes, endothelial cells, adipocytes, and cancer cells [126–128, 148–150]. Since many cell types express a set of miRNAs, circulating miRNAs released from the cells represent their original source. Therefore circulating miRNAs have diagnostic and prognostic potential. For example, high levels of miR-208b, miR-499, and miR-133a are detected in plasma of myocardial infarction patients [151, 152]. Especially, high levels of miR-133a and miR-208b are significantly associated with the risk of death in acute coronary syndrome [153]. Since these mIRNAs are expressed well in the heart, most of these miRNAs might be released from cardiomyocytes or fibroblasts during myocardial injury or infarction. In plasma of type 2 diabetes patients, many miRNAs including miR-126 are detected in low levels [154]. Vascular miRNAs, such as miR-126, miR17, miR-92a, and miR-155 are significantly lower levels in serum of patients with coronary artery disease compared with healthy subjects [155]. Possible reasons for this reduction of miRNA levels are (1) lack of miRNAs storage and production in vascular cells after dramatic release and activation of vasculature, (2) increased uptake of miRNAs into blood cells or into atherosclerotic lesions, or (3) decrease of nSMase activity in blood vessels. 

### 4.3. Functional Messengers (Figure 4)

Several recent studies show that circulating miRNAs can affect target cells [126–128]. miRNAs released by ECs can regulate the biology of vascular cells, including VSMCs, leukocytes and other ECs.

#### 4.3.1. ECs to Distal ECs: miR-126

Zernecke et al. demonstrated that apoptotic bodies from ECs trigger CXCL12 production in other cells in the vascular wall in a paracrine manner [128]. miR-126 is packaged in ECs derived apoptotic bodies, and directly suppressed a set of genes, including regulator of G protein signaling 16 (RGS16), which is known to negatively regulate CXCR4, the CXCL12 receptor. This upregulation of CXCR4 by miR-126 uptake promotes CXCL12 production through an autoregulatory feedback loop. Transfer of miR-126 enriched apoptotic bodies or even miR-126 itself into the ApoE knockout mice reduces the size of lesions, suggesting that the antiatherosclerotic effect of ECs derived apoptotic bodies is at least partially performed by mIR-126. 

#### 4.3.2. Monocyte to ECs: miR-150

Various stimuli, including LPS, oxidative stress, and advanced glycosylated end-product (AGE), trigger miR-150 release from monocytes in vitro [127]. miR-150 is packaged into 20–400 nm sized MVs and these MVs deliver miR-150 into human cultural ECs and inhibit c-Myb expression [127]. In vivo, miR-150 enriched MVs decreased ECs proliferation after injection into mice. miRNAs represent a novel mechanism for communication between monocyte and ECs. Communication via miRNAs between vascular cells might play a role in inflammatory events leading to atherosclerosis.

#### 4.3.3. ECs to VSMCs: miR-143/145

Kruppel-like factor 2 (KLF2) is a key molecule induced by atheroprotective shear stress, and it regulates a set of genes expressed in ECs described above (see Section 3.6). In vitro, KLF2 expression is upregulated by laminar flow or statin [117]. Hergenreider et al. discovered that physiological shear stress and statin treatment activate expression of the miR-143/145 cluster through KLF2 in ECs [126]. MiR-143 and miR-145 are intergenic miRNAs, which control the VSMCs phenotypic switch, tumorigenesis, and adipocyte differentiation [156–158]. Interestingly miR-143/145 synthesized in ECs are secreted into extracellular vesicles and transported into VSMCs [126]. MiR-143 and miR-145 are highly expressed in VSMCs and heart, not usually in resting ECs [159]. ApoE knockdout mice showed low levels of vascular miR-143/145 [160]. Overexpression of miR-143/145 inhibits neointimal formation in acute vascular injury in rats [159]. In contrast, incomplete differentiation of VSMCs is observed in aortas from miR-143/145 knockout mice [136]. Hergenreider et al. demonstrated that injection of ECs derived MVs containing miR-143/145 reduces the formation of atherosclerotic lesion in ApoE knockout mice [126]. These studies demonstrated that atheroprotective flow increases miR-143/145 expression and secretion, and miR-143/145 can be transferred into VSMCs, preventing dedifferentiation. This elegant work suggests that miRNA mediate communication between ECs and VSMCs. Although the in vitro evidence is compelling, definitive in vivo evidence for intercellular communication through miRNA is still lacking.

## 5. Summary

miRNAs function as fine tuners of various biological processes to maintain homeostasis and play a key role in atherogenesis. miRNAs within ECs and VSMCs and monocytes regulate their proliferation, migration, and inflammatory profile. miRNAs can be released by cells and taken up by vascular cells, modulating their cellular biology. miRNA profiles in the blood of humans provide diagnostic and prognostic information during acute vascular events. The rapid development of RNA chemistry has led to the invention of novel modifications of RNA bases and the synthesis of artificial antisense miRNA or antagomir, which may be used as novel therapeutic tools in the future to manipulate miRNA and control vascular inflammatory diseases.

## Figures and Tables

**Figure 1:**
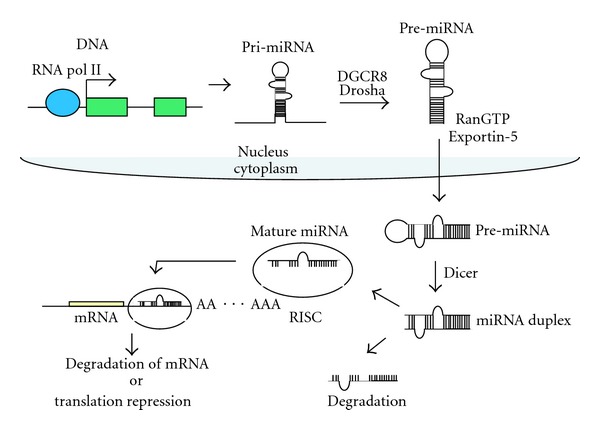
Schema of miRNA biogenesis. Primary miRNA (pri-miRNA) is transcribed by RNA polymerase II. Drosha-DGCR8 complex cleaves pri-miRNA into hairpin-loop structural pre-miRNA. Pre-miRNA is exported to cytoplasm by exportin-5 and RanGTP, then Dicer processes into miRNA duplex. One of the single strands (mature miRNA) is incorporated into RISC and binds to 3′UTR of target mRNA, following translational repression or mRNA degradation.

**Figure 2:**
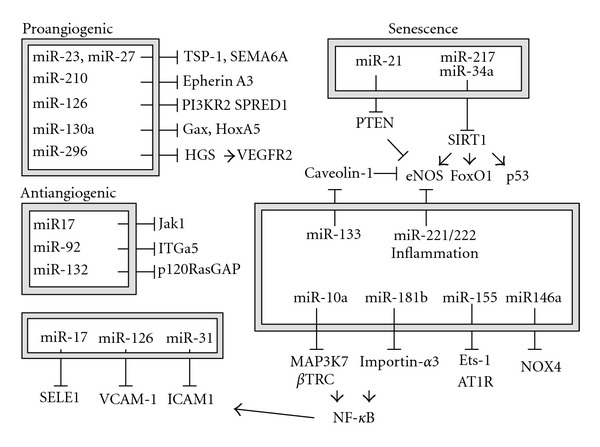
miRNAs regulate ECs functions. Schematic summary of endothelial miRNAs implicated in vascular inflammation. Senescence associated miRNA regulates SIRT1 or PI3K signaling. miRNAs directly involving in inflammatory response regulate angiotensin II signaling, redox signaling, and adhesion molecules. miR-146a suppresses NADPH oxidative subunit NOX4 expression [43]. Proangiogenic miRNAs (miR-23, -27, 210, 126, 130a, 296) and antiangiogenic miRNAs (miR-17, -92, -132) are also important for endothelial homeostasis [44–46].

**Figure 3:**
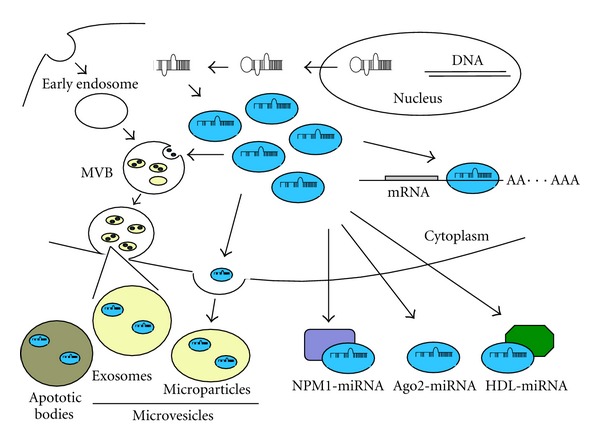
Pathways involved in miRNAs secretion. Extracellular miRNAs (circulating miRNAs) are secreted by incorporation into RNA binding protein, such as Ago2, HDL, and NPM1, or packaged into microvesicles (MVs), including exosomes, microparticles, and apoptotic bodies.

**Figure 4:**
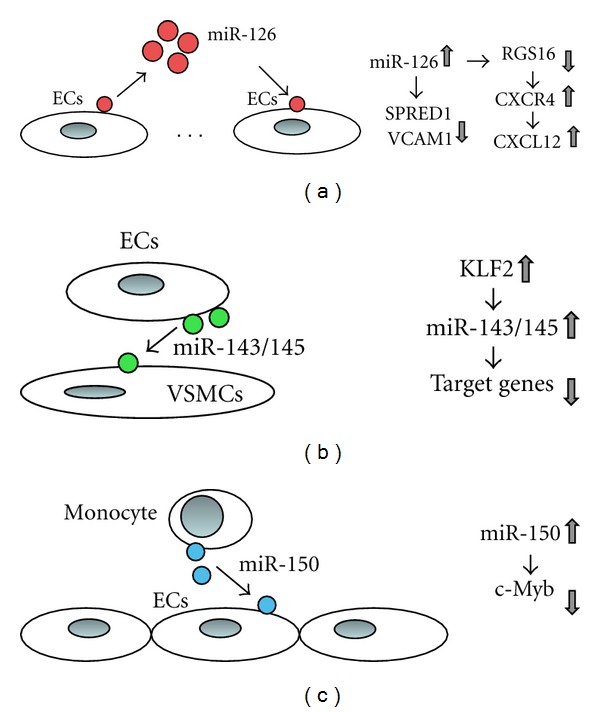
Cell-to-cell communication. ECs are involved in cell-to-cell communication. Schematic representation of three reported communications is shown. (a) ECs-derived apoptotic bodies are transferred to recipient ECs. These apoptotic bodies contain miR-126 that suppress RGS16 expression, enabling CXCR4 to induce CXCL12 production. miR-126 can suppress SPRED1 and VCAM-1 expressions. (b) Shear stress stimulated ECs released miR-143/145 enriched microvesicles, which are transferred into VSMCs. (c) miR-150 is selectively loaded into monocyte derived microvesicles. These microvesicles are transferred to ECs and inhibit c-Myb expression in ECs.
